# Construction, validation, and nitrogen nutrition diagnosis of a critical nitrogen concentration model for cotton

**DOI:** 10.3389/fpls.2026.1776706

**Published:** 2026-03-03

**Authors:** Yukun Qin, Weina Feng, Junying Chen, Cangsong Zheng, Yingshu Leng, Lixian Wang, Lijuan Zhang, Taili Nie

**Affiliations:** 1 Jiangxi Provincial Key Laboratory of Plantation and High-Valued Utilization of Specialty Fruit Tree and Tea, Jiangxi Economic Crops Research Institute, Jiujiang, China; 2 State Key Laboratory of Cotton Bio-Breeding and Integrated Utilization, Institute of Cotton Research, Chinese Academy of Agricultural Sciences, Anyang, China

**Keywords:** critical nitrogen concentration, nitrogen application, nitrogen nutrition diagnosis, nitrogen use efficiency, rapeseed cotton rotation

## Abstract

**Introduction:**

Based on continuous field experiments in Jiujiang City, Jiangxi Province, a critical nitrogen concentration dilution curve model for different parts of cotton in the rapeseed cotton rotation pattern in the Yangtze River Basin was constructed. The nitrogen nutrient index (NNI) was used to diagnose the nitrogen abundance and deficiency status, providing a basis for precision fertilization management in cotton fields.

**Methods:**

A randomized block design was used, and six nitrogen application treatments (pure nitrogen applications at 0, 60, 120, 180, 240, and 300 kg ha*^−^*^1^) were set up, represented as N0, N60, N120, N180, N240, and N300, respectively. The dry matter mass and nitrogen content of each organ of cotton were measured during the initial flowering stage, peak flowering stage, peak boll stage, and boll opening stage in 2023 and 2024, respectively. A critical nitrogen concentration dilution curve model based on the whole plant nitrogen content and the reproductive organ nitrogen content was constructed using the 2023 data. The model was validated using the 2024 data to compare the nitrogen nutrition diagnostic effects of the two models.

**Results:**

The results showed that the critical nitrogen concentration dilution curve models based on cotton whole plant and reproductive organ biomass (represented by *y*_1_ and *y*_2_, respectively) were constructed as follows: *y*_1_ = 26.936*x*^−0.284^ (*R*^2^ = 0.6800, *P* < 0.05), *y*_2_ = 23.504*x*^−0.226^ (*R*^2^ = 0.6226, *P* < 0.05), the RMSE values were 2.76 and 3.53 respectively, with nRMSE values of 15.10% and 14.85% (both within the range of 10% to 20%), indicating that the model accuracy had reached a good level or above. The nitrogen nutrition index based on the whole plant and reproductive organs (represented by NNI_1_ and NNI_2_, respectively) showed a significant positive correlation with seed cotton yield (*P* < 0.05), and a “linear+plateau” relationship with relative yield (RY) [RY = 1.4879NNI_1_ − 1.0095 (*R*^2^ = 0.6434^*^), RY = 0.9461NNI_2_ − 0.3723 (*R*^2^ = 0.6165^*^)]. When the two NNIs reached 1.22 and 1.17, respectively, RY reached its peak. When nitrogen application reached 240 kg ha*^−^*^1^, both NNI values could stably reach 1 or above from the initial flowering stage to the boll opening stage and showed significant advantages in multiple dimensions such as biomass accumulation, yield performance, fiber quality, and nitrogen fertilizer use efficiency.

**Discussion:**

The critical nitrogen concentration dilution curve model based on the reproductive organ nitrogen content and the nitrogen nutrition diagnosis model had the advantages of being more labor-saving and efficient while maintaining the same level of accurate diagnosis, which could provide practical reference for precision fertilization in cotton fields.

## 1 Introduction

Cotton is one of the important cash crops in China. The yield and quality of cotton have an important impact on farmers’ income and the development of the textile industry (Srikala et al., 2023). Nitrogen is the key nutrient for cotton plant growth, development, and yield formation. Reasonable nitrogen application can effectively improve cotton growth and drive the economic benefits of cotton planting. Therefore, mastering mature nitrogen fertilizer application techniques has become an important foundation for guiding high-yield and high-quality cotton production (Pabuayon et al., 2021). However, there is often an issue of excessive fertilizer application in production, which not only increases economic burden but also causes environmental pollution, contrary to the goal of increasing yield (Singh et al., 2025). Economically, excessive nitrogen application increases the cost of nitrogen fertilizer procurement and the input of labor and machinery. The diminishing marginal benefits of nitrogen fertilizer lead to a decrease in cotton planting efficiency for farmers (Charul et al., 2024). In terms of environment, excessive exogenous nitrogen is prone to leaching and runoff with rainfall, causing non-point source pollution in farmlands and air and water pollution through ammonia volatilization, nitrification, and denitrification, which restricts the green development of the cotton industry (Liu et al., 2024). In recent years, the nitrogen use efficiency of cotton production in China has continued to be lower than that in developed countries, and the problem of unreasonable application of nitrogen fertilizer in cotton planting areas in the Yangtze River Basin has become increasingly prominent. Nitrogen use efficiency is only approximately 30%, while the cost of chemical fertilizer has continued to rise, which has seriously affected the improvement of agricultural production efficiency, quality, and economic benefits (Liu et al., 2022; Qin et al., 2025b). Since the implementation of the “double reduction” policy in China, a scientific and reasonable nitrogen supply is of great significance to the input reduction, yield increase, quality improvement, and efficiency increase in the cotton planting industry (He et al., 2025). In recent years, the rapeseed cotton rotation mode in the Yangtze River Basin has experienced the exploration and development to the stage of gradual promotion, maintaining an annual planting area of more than 70,000 hectares, which has broad prospects for improving the land utilization rate, enhancing the enthusiasm of farmers, and improving the benefits of cotton planting (Wang et al., 2022). It has become the key focus of the current research on light, simple, and efficient agricultural production to accurately and quantitatively regulate the nitrogen application link of rape cotton rotation mode and improve the accuracy of the nutrient abundance and deficiency diagnosis model.

The application of sufficient nitrogen fertilizer was beneficial to the dry matter accumulation of vegetative organs in the middle stage of cotton, but excessive nitrogen fertilizer application affected the material accumulation performance of cotton in the key period of vigorous development of reproductive organs, such as boll setting and boll opening stage. Adverse factors such as excessive development of vegetative organs, mutual shading of plant groups, and late maturing of leaves could lead to the obstruction of boll synthesis and maturation in the later stage (Zhang et al., 2021). Relevant studies showed that the Xinjiang cotton region could build a reasonable cotton population structure by reducing nitrogen application, increasing the number of bolls per plant and yield, and improving the fertilizer use rate (Pei et al., 2023). Increasing nitrogen fertilizer application in Northwest China could improve the yield of drip irrigated cotton, but the nitrogen uptake and fiber quality had not been significantly increased (Hou et al., 2021). Quantitative diagnosis and evaluation of nitrogen nutrition status of cotton plants and the construction of practical, accurate, and mature nitrogen nutrition diagnosis tools could provide a reference for accurately identifying whether cotton plants were in the state of nitrogen surplus or nitrogen loss and had practical significance for guiding field production (Chen et al., 2022; Hadisseh et al., 2021).

In order to master the situation of cotton nitrogen quality, the method of determining plant nitrogen by laboratory test was often used to analyze and determine the limited and unrestricted state of plant nitrogen demand. However, this method was time-consuming, expensive, inefficient, and lagging. These adverse factors would reduce its utilization value in the field of rapid detection and diagnosis scenarios, which was not conducive to the promotion and application of light, simplified, and efficient cultivation and production mode (Mojtaba et al., 2022). The diagnosis technology of nitrogen nutrition index based on the critical nitrogen dilution curve (CNDC) model, by constructing the quantitative response relationship between biomass and critical nitrogen concentration, has progressive features such as accurate quantitative analysis, dynamic real-time monitoring, and consideration of yield effects and ecological effects compared with conventional analysis methods. Moreover, the constructed critical nitrogen concentration model of cotton reflects the local actual situation and improves the accuracy of nutrition diagnosis (Habibi et al., 2024; Deng et al., 2023; J. et al., 2023). The study showed that the critical nitrogen concentration model based on drip irrigation cotton field in southern Xinjiang was Nc = 3.91 × DM^−0.24^ (*R*^2^ = 0.906), and the nutritional diagnosis results showed that 240–360 kg·ha^−1^ of nitrogen application was the appropriate nitrogen application rate for drip irrigation cotton (Ma et al., 2018). Based on the aboveground biomass of cotton plants in the Huang Huai region, the critical nitrogen concentration dilution curve models of two conventional varieties were Nc = 3.037 × DM^−0.098^ (*R*^2^ = 0.984) (Xue et al., 2008) and Nc = 2.49 × DM^−0.12^ (*R*^2^ = 0.649) (Feng et al., 2022), respectively. The diagnostic results showed that nitrogen application of more than 360 kg·ha*^−^*^1^ was not conducive to yield improvement. Most of the above studies were concentrated in the Xinjiang cotton region and the Yellow River Basin cotton region, and most of the models were established for the dry matter accumulation of complete plants or crops in the early and middle stages of growth. However, for the Yangtze River Basin cotton region, which is one of the three major cotton regions, current reports mostly focused on the early and middle growth stages of cotton from the seedling stage to the flowering and boll stage and lacked observation and research on the characteristics of the boll opening stage. Secondly, the whole cotton plant was mostly taken as the research object, and there was a lack of analysis on the construction model of reproductive organs. Thirdly, the Yangtze River Basin belongs to a subtropical monsoon climate, with strong interannual rainfall and temperature fluctuations. Current studies were mostly short-term test data, and there was a lack of multiyear fixed-point tests to reflect the impact of climate interannual change on model parameters.

Therefore, in this study, a 2-year continuous field fixed-point experiment was carried out, and six nitrogen application levels were set up, aiming to build a critical nitrogen dilution curve model based on the whole plant and reproductive organs of cotton under the rapeseed cotton rotation mode, and to diagnose and evaluate the nitrogen nutrition status of cotton in the key growth stage in order to provide quantitative indicators for cotton growth and yield performance. This study was conducted in order 1) to determine the critical nitrogen dilution curve based on two parts of cotton plant (whole plant and reproductive organ) and evaluate the simulation effect; 2) to quantify the relationship between the nitrogen nutrition index (NNI) and nitrogen uptake, yield, yield components, and nitrogen use efficiency; and 3) to formulate the precise nitrogen application scheme for rapeseed cotton rotation cotton based on the diagnosis results of nitrogen nutrition, cotton yield, and nitrogen use efficiency. This study could provide new insights for the diagnosis of nitrogen efficiency in cotton fields in the Yangtze River Basin.

## 2 Materials and methods

### 2.1 Site description

The experimental site was located at the Chaisang Experimental Base of the Provincial Economic Crop Research Institute in Jiujiang City, Jiangxi Province (30°05′N, 116°54′E), with a subtropical monsoon climate, an average altitude of 32 m, an average frost-free period of 251 days per year, and an average annual precipitation of 1,410 mm from 2002 to 2022. The soil texture was loamy lake alluvial soil, of the red soil type. Before sowing in 2023, the pH value of the cultivated soil in the 0–20-cm layer was 7.60, the organic matter content was 9.77 g kg^−1^, the available phosphorus content was 30.27 g kg^−1^, and the available potassium content was 188.40 g kg^−1^.

### 2.2 Experimental design

This experiment was based on the field continuous positioning experiment of different fertilization rates under the rapeseed cotton rotation mode starting in 2018. The data obtained from the cotton season from May to November 2023 and from May to November 2024 after 5 years of continuous positioning were used. A single-factor randomized block design was used in the experiment. Six treatments with different nitrogen application rates were set up, and 60 kg ha^−1^ was used as the nitrogen concentration gradient. The nitrogen application rates of each treatment were set at 0, 60, 120, 180, 240, and 300 kg ha^−1^, represented as N0, N60, N120, N180, N240, and N300, respectively. The test variety was Jinghua cotton 116, which was provided by Jiangxi Institute of Economic Crops. Each process was provided with four repeating plots, with a length of 17.5 m and a width of 2.28 m, and a total of 24 test plots. In the plot, the row spacing of cotton was 0.76 m, the plant spacing was 0.16 m, and the sowing density and the actual density were 82,500 plants·ha^−1^. The application rates of phosphorus and potassium fertilizers were the same in all treatments. The types of phosphorus fertilizer (containing P_2_O_5_ 12%) and potassium fertilizer (containing K_2_O 60%) were calcium superphosphate and potassium chloride, respectively. The application rates were 90 and 180 kg ha^−1^, respectively; nitrogen fertilizer (46.4% of pure nitrogen) was urea, which was applied at a ratio of 4:6 at the budding stage (13 June 2023, 21 June 2024) and the flowering and bolling stage (15 July 2023, 15 July 2024) every year. Phosphorus and potassium fertilizers were applied at one time at the budding stage every year. The fertilization method was unified as manual application. Cotton field direct seeding was conducted on 18 May 2023 and 17 May 2024. On 20 October 2023 and 18 October 2024, rapeseed was sown in the cotton forest in the experimental area. The cotton and rapeseed straw were not returned to the field, and the chemical control weeding and other management measures were the same as those in the local field.

### 2.3 Measurements

#### 2.3.1 Accumulation of dry matter in cotton

In 2023 and 2024, three cotton plants (including roots) with complete disease-free, basically consistent growth and continuous arrangement were taken from each plot at the emergence stage, five-leaf stage, budding stage, initial flowering stage, peak flowering stage, peak boll stage, boll opening stage, and harvest stage of cotton in 2023 and 2024 as representative samples. After washing, they were divided into vegetative organs (roots, stems, and leaves) and reproductive organs (buds, flowers, and bolls before the boll opening stage, seed cotton and boll shell during the boll opening stage). The plants were heated in an oven at 105°C for 30 min, dried at 80°C to a constant weight, and then weighed to calculate the dry matter accumulation per plant.

#### 2.3.2 Nitrogen content of cotton vegetative and reproductive organs

The samples of vegetative and reproductive organs taken every year at the initial flowering stage, peak flowering stage, peak boll stage, and boll opening stage were crushed, and the total nitrogen content was determined by the Kjeldahl method (Quirino et al., 2023). Firstly, the reproductive period sample data for constructing the two models should be consistent and cover the entire reproductive stage as much as possible to improve the accuracy of the models. Secondly, in order to save labor and material costs and facilitate the promotion and application of research results, this experiment chose to measure the nitrogen content of representative growth stage cotton plants to construct a CNDC model. Thirdly, there was little difference in nutrient content among cotton plants during the emergence, five-leaf, and budding stages, and reproductive organs only began to form after the budding stage. Therefore, the selected growth stages could be simplified and eliminated. Therefore, the nitrogen content of vegetative and reproductive organ samples was ultimately determined during the initial flowering stage, peak flowering stage, peak boll stage, and boll opening stage.

#### 2.3.3 SPAD value and nitrogen content of cotton functional leaves

Ten continuous cotton plants with basically the same growth trend were marked in each plot on 15 July 2023 and 2024, respectively, and the soil and plant analysis development (SPAD) value and leaf nitrogen content of cotton functional leaves (i.e., the last four leaves) were measured by a plant nutrient analyzer (produced by Shijiazhuang Fansheng Company, Shijiazhuang, Hebei, China, model ZZY-A) on 15 July, 1 August, 15 August, 30 August, 15 September, and 30 September. Chlorophyll has a strong absorption of 650 nm red light and weak absorption of 940 nm infrared light. By calculating the ratio of the transmittance coefficients of the two wavelengths, the relative quantity of chlorophyll (i.e., SPAD value) could be determined. Based on near-infrared spectroscopy technology, the strong correlation between nitrogen and chlorophyll was utilized to invert leaf nitrogen content through chlorophyll content.

#### 2.3.4 Cotton yield and its constituent factors

Fifty mature cotton bolls were collected in each plot on 15 October 2023 and 10 October 2024 and weighed after indoor air drying. At the same time, all boll numbers of 10 continuous cotton plants with complete disease-free and consistent growth were obtained, and the boll density was calculated. All seed cottons in the plots were harvested, tested, and weighed by ginning to obtain the yield of lint and calculate the lint percentage (lint percentage was the ratio of lint yield to seed cotton yield) (John et al., 2021).

#### 2.3.5 Cotton fiber quality

The fiber quality of cotton boll samples collected in each plot was determined, including the average length of the upper half, breaking strength, uniformity index, Micronaire value, and elongation (Carlos et al., 2021).

### 2.4 Calculations

#### 2.4.1 Population nitrogen uptake

The product of the dry matter accumulation, measured nitrogen concentration, and actual harvest density of each cotton plant’s vegetative and reproductive organs was the population’s nitrogen uptake, with the unit of kg·ha*^−^*^1^.

#### 2.4.2 Critical nitrogen concentration dilution curve

The critical nitrogen concentration dilution curve model was constructed based on the test data in 2023: 1) the cotton population biomass under different nitrogen application rates was compared and analyzed, and the biomass of each treatment group (divided into the whole plant, vegetative organ, and reproductive organ population biomass) was analyzed by significant variance, and each treatment group was divided into two groups: the nitrogen-limited group and the non-limited group, according to the following: the limited group was the treatment in which the population biomass increased significantly with the increase of nitrogen application rate, and the non-limited group was the treatment in which the biomass did not increase significantly with the increase of nitrogen application rate). 2) For the limited group, the population biomass and nitrogen concentration were linearly fitted; for the unrestricted group, the vertical coordinate of the intersection of the mean of the population biomass and the linear equation was taken as the critical nitrogen concentration of the sample. 3) The critical nitrogen concentration dilution curve model was constructed according to the critical nitrogen concentration value of each treated sample and the population biomass. The specific calculation formula is shown in Equation 1:

(1) $$N_{c}=a_{c}\cdot DW^{-b}$$

Where *N_c_* is the critical nitrogen concentration of cotton (g·kg^−1^), *a_c_* (g·kg^−1^) is the nitrogen concentration when cotton dry matter reached 1 t·ha^−1^, *DW* (t·ha^−1^) is the dry matter accumulation of cotton, and *b* is the slope of the critical nitrogen dilution curve (Zhou et al., 2025).

#### 2.4.3 Relevant parameters of the dilution curve at the critical nitrogen concentration

The root mean square error (RMSE) of the calculation model and the normalized root mean square error (nRMSE) were calculated. The fitting effect and reliability of the critical nitrogen concentration model were analyzed by establishing a 1:1 linear scatter plot between the simulated value and the measured value. The correlation coefficient *R*^2^ of the dilution curve equation of the critical nitrogen concentration was calculated, and the reliability of the model was analyzed. The calculation formulas were as follows in Equations 2–4:

(2) $$RMSE=\sqrt{\left[\sum_{i=1}^{n}\frac{{\left(P_{i}-O_{i}\right)}^{2}}{n}\right]}$$

(3) $$nRMSE=\sqrt{\left[\sum_{i=1}^{n}\frac{{\left(P_{i}-O_{i}\right)}^{2}}{n}\right]}\times \frac{100}{\bar{O}}$$

(4) $$R^{2}=\frac{{\left[\sum_{i=1}^{n}\left(P_{i}-\bar{P})(O_{i}-\bar{O}\right)\right]}^{2}}{\sum_{i=1}^{n}{\left(P_{i}-\bar{P}\right)}^{2}\sum_{i=1}^{n}{\left(O_{i}-\bar{O}\right)}^{2}}$$

Where *RMSE* is the root mean square error, *nRMSE* is the normalized root mean square error after standardization, and *P_i_* is the simulated value of nitrogen concentration in 2024 for each treatment. *O_i_* is the measured value of nitrogen concentration of each treatment in 2024, $\bar{O}$ is the average value of the measured nitrogen concentration, and *n* is the number of samples, which was 72 in this test. The RMSE was used to measure the average difference between the simulated value and the measured value. The smaller the RMSE value, the higher the consistency between the simulated value and the measured value, and the smaller the deviation. On the contrary, the nRMSE was used to compare the model performance of different unit data. An nRMSE <10% indicated that the simulation effect of this model was excellent, 10% < nRMSE < 20% indicated good performance, 20% < nRMSE < 30% indicated general performance, and nRMSE > 30% indicated poor performance. The closer the *R*^2^ value was to 1, the higher the reliability was (Qiang et al., 2019; Bai et al., 2024).

#### 2.4.4 Nitrogen nutrition index based on the critical nitrogen concentration dilution curve

The NNI for each treatment was calculated based on simulated critical nitrogen concentration values and measured nitrogen concentration values. The specific calculation formula is shown in Equation 5:

(5) $$NNI=N_{t}/N_{c}$$

Where *NNI* is the nitrogen nutrition index, *N_t_* is the measured value of nitrogen concentration, and *Nc* is the simulated value of nitrogen concentration calculated based on the dilution curve model of critical nitrogen concentration. NNI could directly reflect the nitrogen nutrition status of crops, NNI = 1. The results showed that the nitrogen nutrition of the plant was suitable: NNI >1 was the state of plant nitrogen surplus, and NNI <1 was the state of plant nitrogen deficiency (Lu et al., 2024).

#### 2.4.5 Nitrogen fertilizer use efficiency

Nitrogen fertilizer use efficiency was calculated based on parameters such as biomass, seed cotton yield, lint percentage, nitrogen uptake, nitrogen application rate, and nitrogen concentration for each treatment in 2023 and 2024. The relevant formulas were as follows in Equations 6–12:

Population nitrogen uptake 

(6) NU=population biomass×measured nitrogen concentration

Nitrogen agronomic efficiency  

(7) $$NUEa=(Y_{n}-Y_{0})/F_{n}$$

Nitrogen internal use   efficiency 

(8) $$NUEi=Y_{n}\times LP/NU$$

Nitrogen physiological use efficiency 

(9) $$NUEp=(Y_{n}-Y_{0})/(NU_{n}-NU_{0})$$

Nitrogen fertilizer recovery rate 

(10) $$AREN=(NU_{n}-NU_{0})/F_{n}$$

(11) $$\text{Harvest index }HI=Y_{n}/\text{population biomass}\times 100\%$$

Nitrogen partial factor productivity 

(12) $$NPFP=Y_{n}/F_{n}$$

where *NU* is the population nitrogen uptake, *Y_n_* is the seed cotton yield in the nitrogen application plots, *Y*_0_ is the seed cotton yield of non-nitrogen application plots, *F_n_* is the nitrogen application rate, *LP* is the lint percentage, *NU_n_* is the nitrogen uptake rate of the nitrogen fertilizer application plots, and *NU*_0_ is the nitrogen uptake rate in the non-nitrogen application plots (Ma et al., 2023; Li et al., 2017).

NUE_a_ reflects the net yield increase effect of nitrogen application guided by the CNDC model; AREN can directly reflect the proportion of nitrogen absorbed by crops, and it is the core index to judge whether nitrogen supply matches crop demand; NPFP and HI are economic indicators concerned by farmers, reflecting the economic output of unit nitrogen application and biomass; and NUE_i_ and NUE_p_ reveal the transformation from nitrogen uptake to yield from the physiological level. Joint analysis of various nitrogen use efficiency indicators can comprehensively evaluate the diagnostic application effect of the CNDC model.

#### 2.4.6 Yield stability and sustainability index

The stability and sustainability of cotton production based on the actual seed cotton yield in 2023 and 2024 were calculated. The calculation formulas were as follows in Equations 13, 14:

(13) $$SI=\;\frac{SD}{Y}$$

(14) $$SYI=\frac{\left(Y-SD\right)}{Ymax}$$

Where *SI* is the yield stability index, *SYI* is the yield sustainability index, *Y* is the average yield, *SD* is the standard deviation of yield between treatments, and *Y_max_* is the maximum yield (Qin et al., 2025b).

#### 2.4.7 Economic nitrogen application rate

Based on the simulation of seed cotton production in 2023 and 2024, a quadratic curve equation of seed cotton production with nitrogen application rate was obtained. Combined with parameters such as fertilizer price, labor cost, and seed cotton purchase price (pure nitrogen price of 8.3 yuan kg^−1^, urea price of 3.8 yuan kg^−1^, seed cotton purchase price of 5.8 yuan kg^−1^, and seed cotton manual harvesting cost of 2.6 yuan kg^−1^), the optimal economic nitrogen application rate was calculated by simulating the economic benefits (in this study, the economic benefits were equal to the total purchase price of seed cotton per unit area minus seed cotton labor cost minus urea cost).

### 2.5 Data analysis

Excel 2010 was used for data organization. Origin 2021 was used to draw correlation heat maps, box plots, and other charts, and SPSS 19.0 was used for analysis of variance.

## 3 Results

### 3.1 Analysis of temperature and rainfall

**Figure 1:**
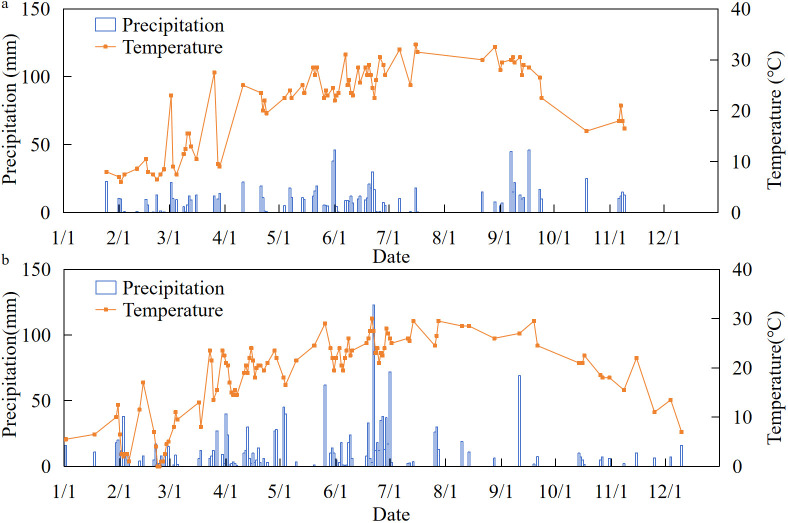
The temperature and precipitation conditions in 2023 and 2024: **(a)** 2023 and **(b)** 2024.

The results in **Figure 1** show that the total annual rainfall in 2023 was 974.9 mm, and there would be high temperatures and drought in August. The total annual rainfall in 2024 was 1,526.7 mm, an increase of 56.60% compared to 2023. Both years showed a common feature of heavy rainfall from May to July and low rainfall and high temperatures from August to October. However, the rainfall from May to July and August to October in 2024 would be higher than the same stage in 2023. The hot and rainy climate, as a stress factor, might increase the probability of cotton bud shedding, hinder the formation of biomass and seed cotton yield in the later stage, and change the distribution ratio of nitrogen between vegetative and reproductive organs. This would cause fluctuations in the CNDC model based on the response relationship between nitrogen nutrition and biomass between the two years, which would have a certain impact on the robustness of the model.

### 3.2 The effect of nitrogen application rate on nitrogen absorption in the cotton population

**Figure 2:**
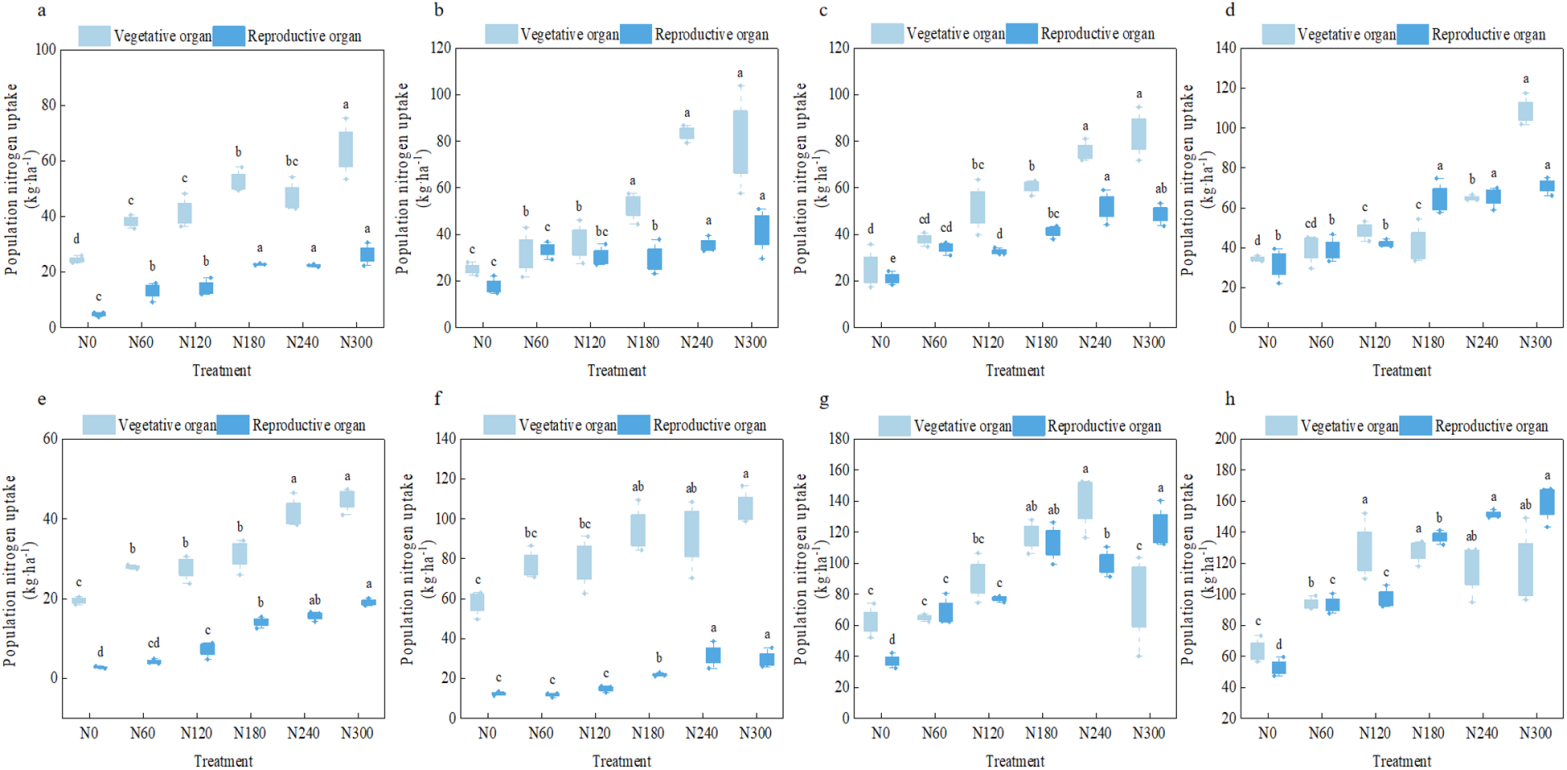
Nitrogen absorption of cotton population under different nitrogen application rates. **(a–d)** The nitrogen absorption of cotton during the initial flowering stage, peak flowering stage, peak boll stage, and boll opening stage in 2023, respectively. **(e–h)** The nitrogen absorption of cotton during the initial flowering stage, peak flowering stage, peak boll stage, and boll opening stage in 2024, respectively.

The results in **Figure 2** indicated that compared with the N0 treatment, the N180~N300 treatments significantly increased the nitrogen absorption of vegetative organs and reproductive organ populations. The nitrogen absorption of vegetative organs during the initial flowering stage, peak flowering stage, peak boll stage, and boll opening stage increased significantly by 19.31%~2,233.84% in 2023 and 58.76%~130.45% in 2024 (excluding the peak boll stage) and 67.56%~437.26% in 2023 and 76.09%~584.07% in 2024. Compared with the N240 treatment, the N180 treatment significantly reduced the nitrogen uptake of reproductive organs by 16.79%, 20.47%, 30.67%, and 9.73% at the peak flowering stage in 2023, peak boll stage in 2023, peak flowering stage in 2024, and boll opening stage in 2023, respectively. However, the nitrogen uptake of reproductive organs in N300 treatment did not increase significantly in 2 years (except for the peak boll stage in 2024). The results showed that compared with no nitrogen application, nitrogen application of 180~300 kg ha*^−^*^1^ significantly improved the nitrogen absorption of cotton, but the nitrogen application of 300 kg ha*^−^*^1^ was not conducive to the significant increase of nitrogen absorption of cotton population compared with 240 kg ha*^−^*^1^, resulting in low marginal nitrogen uptake efficiency.

### 3.3 The effect of nitrogen application rate on SPAD value and nitrogen content of functional leaves in cotton

**Figure 3:**
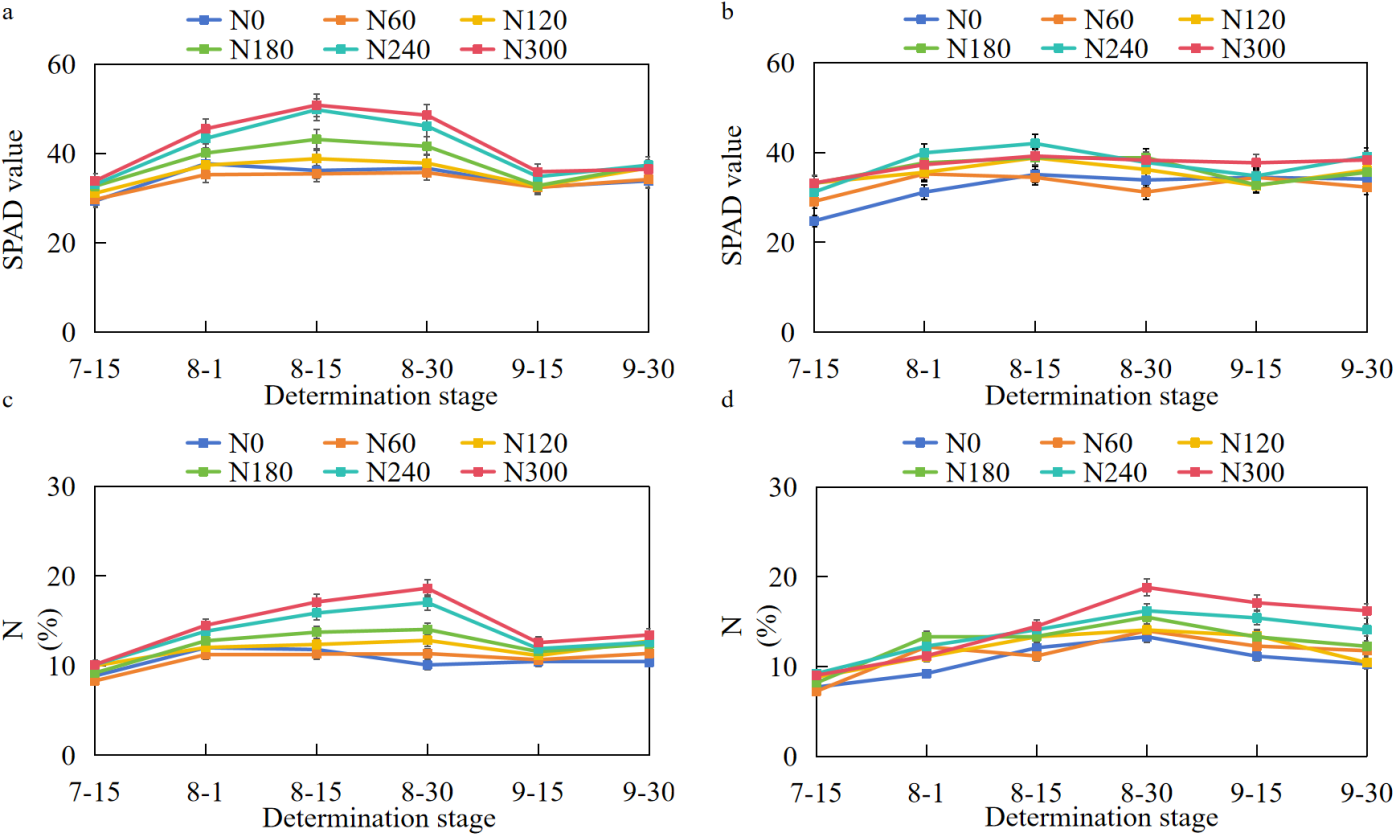
SPAD values and nitrogen contents of cotton inverted four leaves under different nitrogen application rates. **(a, b)** SPAD values of cotton leaves in 2023 and 2024, respectively; **(c, d)** nitrogen content of cotton leaves in 2023 and 2024, respectively.

The results in **Figure 3** indicated that the SPAD values of functional leaves under various treatments generally showed a gradual upward trend from 15 July (initial flowering stage) to 15 August (flowering and boll forming stage) and then a gradual downward trend with fluctuations from 15 August to 30 September. The nitrogen (N) content gradually increased from 15 July (initial flowering stage) to 30 August (peak boll stage) and then gradually decreased from 30 August to 30 September. Both the SPAD values and nitrogen content of functional leaves under the N0, N60, and N120 treatments were relatively low, while the SPAD values of functional leaves under the N240 and N300 treatments were higher than those of the other treatments from 15 July to 30 September. This result indicated that nitrogen application could enhance the SPAD values and nitrogen content of functional leaves, which was beneficial for the synthesis and accumulation of dry matter.

### 3.4 Construction and validation of the dilution curve model for critical nitrogen concentration in cotton

**Figure 4:**
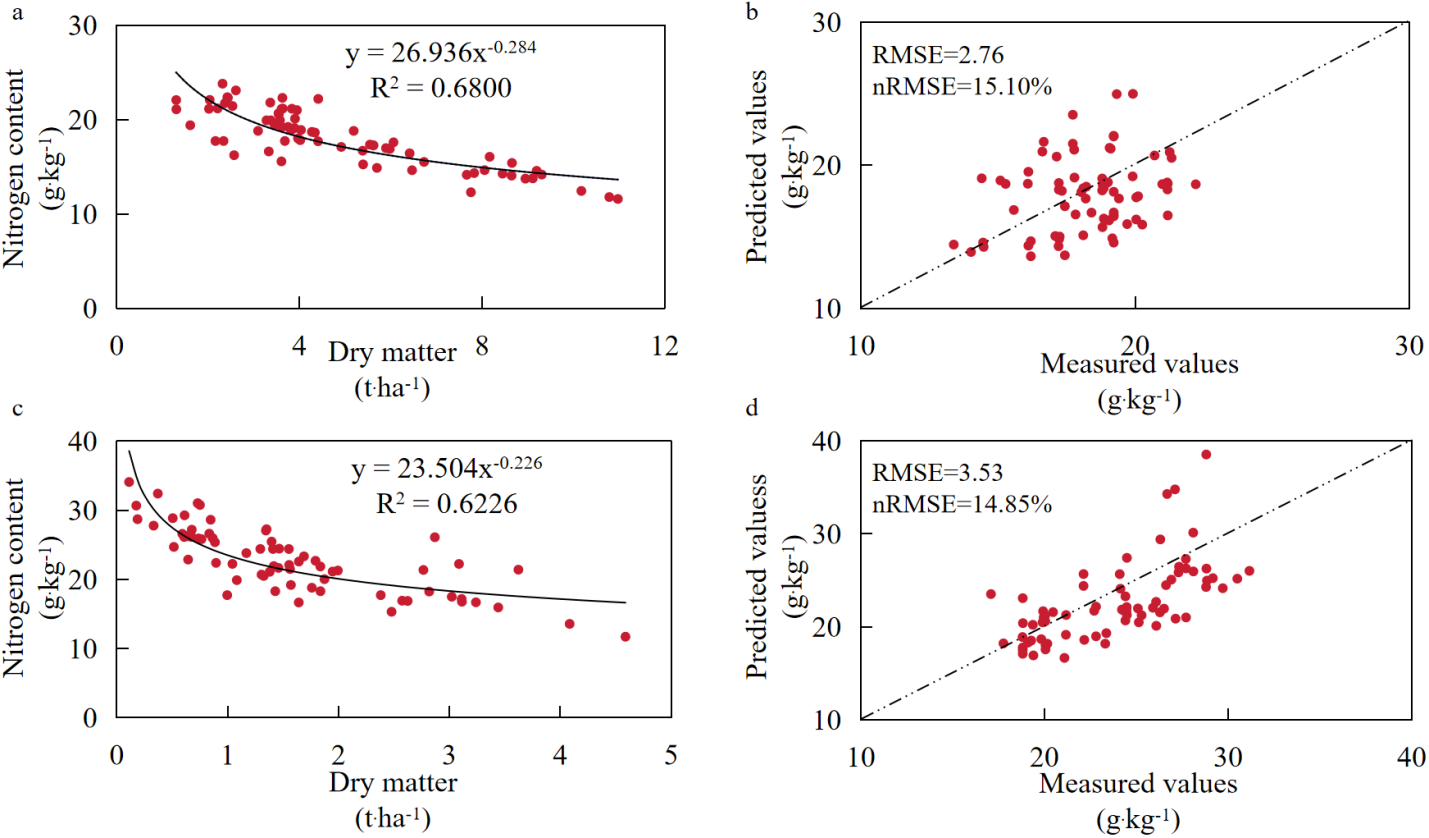
Model construction and validation of the cotton critical nitrogen concentration dilution curve. **(a, b)** The dilution curves of critical nitrogen concentration for cotton based on the whole plant nitrogen concentration in 2023 and the model validation based on the 2024 data. **(c, d)** The dilution curves of critical nitrogen concentration for cotton based on the reproductive organ nitrogen concentration in 2023 and the model validation based on the 2024 data, respectively.

The results in **Figure 4** showed that the nitrogen concentration of the whole plant and the reproductive organ of cotton decreased with the increase of dry matter. The critical nitrogen concentration dilution curve model based on the whole plant nitrogen concentration was *y* = 26.936*x*^−0.284^ (*R*^2^ = 0.6800, *P* < 0.05), and the critical nitrogen concentration dilution curve model based on the reproductive organ nitrogen concentration was *y* = 23.504*x*^−0.226^ (*R*^2^ = 0.6226, *P* < 0.05). The measured results in 2024 were brought into the two models for validation, and the validation parameters RMSE and nRMSE were 2.76 and 3.53 and 15.10% and 14.85%, respectively. The validation results showed that the simulation effects of the two models were above a good level (both in the range of 10%~20%). The critical nitrogen concentration dilution curve model based on the whole plant nitrogen concentration and reproductive organ nitrogen concentration could be used to estimate the cotton plant nitrogen concentration in the rapeseed cotton rotation mode and achieve a good prediction effect. In contrast, the construction of the critical nitrogen concentration models based on reproductive organs and subsequent nitrogen nutrition diagnosis in field operations reduced labor and yield damage (without damaging the entire plant, only representative samples of reproductive organs were taken), reduced diagnostic errors (nRMSE reduced from 15.10% to 14.85%), and had more efficient, convenient, and economical advantages. Although interannual climate variability could affect the parameters of the CNDC model, the years 2023 and 2024 exhibited a common characteristic of heavy rainfall from May to July and low rainfall and high temperatures from August to October. Under these conditions, the fitted model *R*^2^ reached 0.6 or above. Although the accuracy was limited, it still demonstrated the good applicability of the CNDC model constructed in this study.

### 3.5 Nitrogen nutrition diagnosis based on the critical nitrogen concentration dilution curve

**Figure 5:**
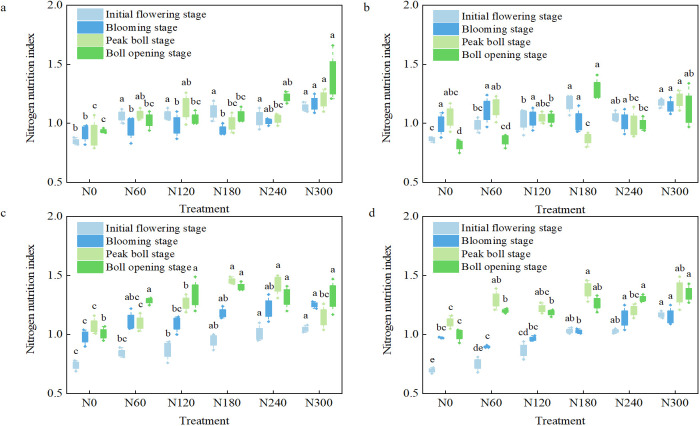
Dynamic changes in nitrogen nutrient index of cotton at various growth stages. **(a–d)** The changes in cotton nitrogen nutrition diagnostic index based on whole plant nitrogen concentration in 2023, reproductive organ nitrogen concentration in 2023, whole plant nitrogen concentration in 2024, and reproductive organ nitrogen concentration in 2024, respectively.

The results in **Figure 5** indicated that in 2023 and 2024, the NNI based on the whole plant (represented by NNI_1_) and the NNI based on reproductive organ (represented by NNI_2_) during the initial flowering stage, peak flowering stage, peak boll stage, and boll opening stage of N0 treatment were both below 1, indicating that not applying nitrogen would cause cotton plants to be in a nitrogen-deficient state for a long time. As the nitrogen application rate increased, the NNI_1_ of each treatment from the initial flowering stage to the opening stage showed an overall upward trend. The NNI_1_ values of the N60, N120, and N180 treatments from the initial flowering stage to the opening stage in 2023 were within the ranges of 0.97–1.07, 0.98–1.13, and 0.94–1.10, respectively. In 2024, the NNI_1_ values were within the ranges of 0.84–1.29, 0.87–1.34, and 0.95–1.45, respectively. This indicated that cotton plants could not reach a stable nitrogen suitable state within the nitrogen application range of 60 to 180 kg ha^−1^. Two years of N240 and N300 treatments resulted in NNI_1_ exceeding 1 in each stage, and 2 years of N300 treatment resulted in NNI_2_ exceeding 1. Two years of N240 and N300 treatments did not show significant differences in NNI_1_ and NNI_2_ during the initial flowering stage, peak flowering stage, and boll opening stage (*P <* 0.05). This phenomenon indicated that cotton plants with nitrogen application of 240–300 kg ha^−1^ could stably reach a nitrogen suitable state at various growth stages, and even nitrogen luxury absorption had occurred. Increasing the nitrogen application rate from 240 to 300 kg ha^−1^ was not conducive to the effective improvement of NNI, resulting in the waste of nitrogen resources. The NNI index of cotton in 2024 fluctuated significantly and was directly related to the stage differences in rainfall and temperature during the growth stage. On the one hand, the hot and rainy climate conditions from May to July significantly increased the rate and effectiveness of soil nitrogen mineralization and promoted the absorption and utilization of nitrogen by cotton roots, and the increase in plant nitrogen accumulation effectively enhanced the NNI index level. On the other hand, compared with 2023, the climate characteristics from August to October 2024 were as follows: rainfall was concentrated within a few days, followed by persistent drought. The drought stress of low rainfall and high temperature inhibited the root activity and nitrogen absorption capacity of cotton, increased the flower bud shedding rate, and changed the distribution ratio of nitrogen between nutritional growth and reproductive growth. These factors all increased the fluctuation amplitude of reproductive organ NNI.

### 3.6 The effect of nitrogen application rate on cotton yield and yield stability

**Figure 6:**
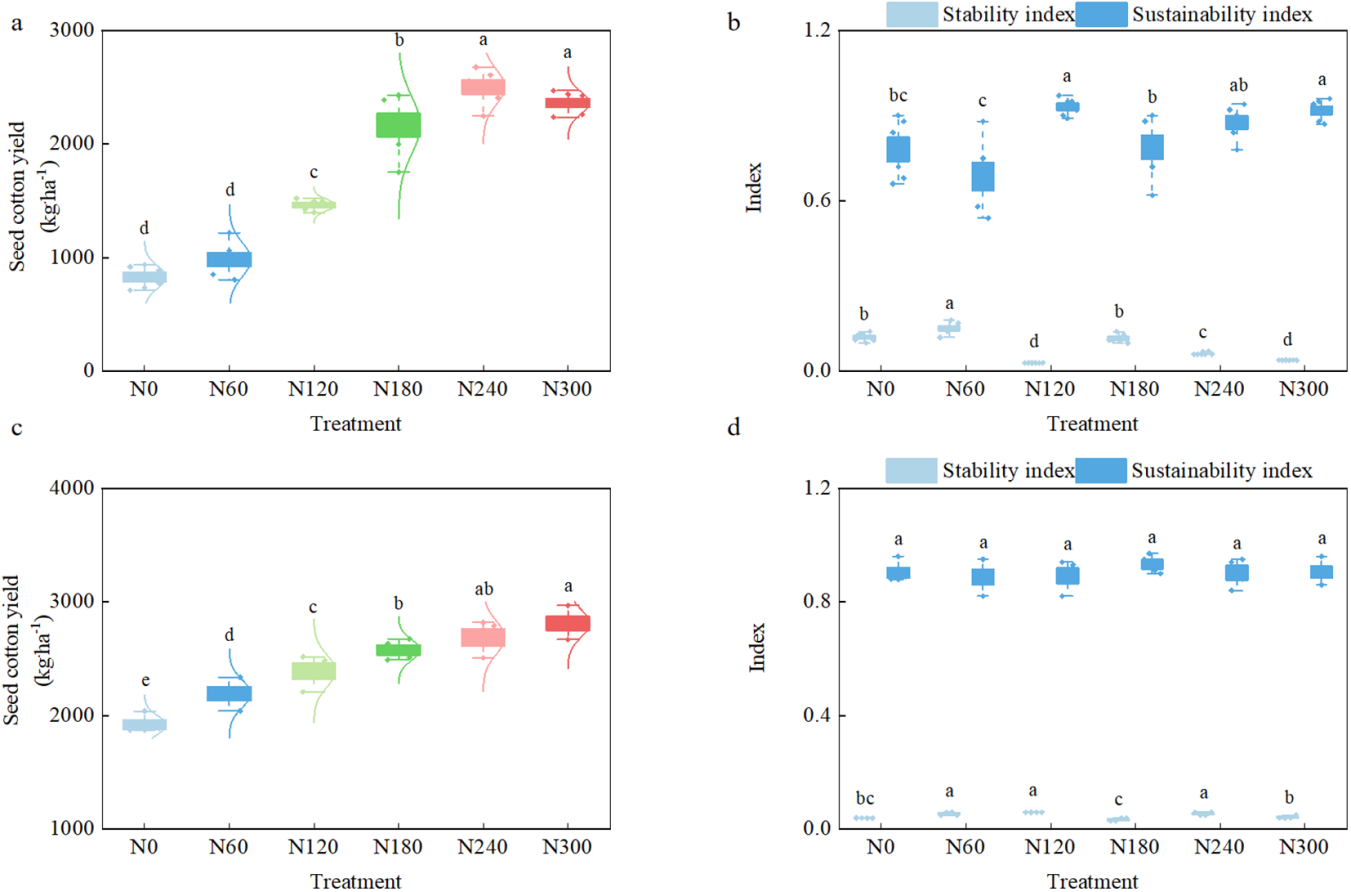
Seed cotton yield, yield stability, and sustainability index under different nitrogen application rates. **(a, c)** Seed cotton yield in 2023 and 2024; **(b, d)** yield stability and sustainability index in 2023 and 2024 for each treatment.

The results in **Figure 6** indicated that there was no significant difference in seed cotton yield between the N240 and N300 treatments in 2023 and 2024, but they were significantly increased compared to the N0, N60, and N120 treatments. The significant increase in 2023 was 61.33% to 201.89%, and the significant increase in 2024 was 12.25% to 46.54%. Analyzing the yield stability and sustainability index, it was found that although the yield stability index of the N300 treatment decreased significantly by 33.33% and 20.00% compared to the N240 treatment in the past 2 years, there was no significant difference in the yield sustainability index, and there was no significant difference in the yield sustainability index between the N240 treatment and the N120 treatment or the N180 treatment.

The results in **Table 1** showed that the boll density and single boll weight of the N300 treatment were significantly improved compared to the N0, N60, and N120 treatments. In 2023, the boll density increased significantly by 254.64%, 212.52%, and 156.41%, respectively, and the single boll weight increased significantly by 32.72%, 23.63%, and 21.96%, respectively. In 2024, the boll density increased significantly by 61.86%, 51.66%, and 27.75%, respectively, and the single boll weight increased significantly by 19.82%, 15.38%, and 10.35%, respectively. There was no significant difference in boll density and single boll weight between the N0 and N60 treatments or between the N60 and N120 treatments over the course of 2 years. In 2023, the N300 treatment showed significant improvements in boll density and single boll weight compared to the N120, N180, and N240 treatments. Additionally, the N180 treatment showed significant increases in boll density, single boll weight, and lint percentage by 33.84%, 13.85%, and 5.39%, respectively, compared to the N120 treatment. However, in 2024, there were no significant differences in single boll weight and lint percentage between the N300 treatment and the N180 and N240 treatments, and there were no significant differences in boll density and single boll weight between the N180 and N120 treatments.

**Table 1:** Composition factors of cotton yield under different nitrogen application rates.

Year	Treatment	Boll density (bolls m^−2^)	Boll weight (g)	Lint percentage (%)
2023	N0	29.08 e	2.72 d	35.94 d
	N60	33.00 de	2.92 cd	37.35 c
	N120	40.22 d	2.96 c	37.83 c
	N180	53.83 c	3.37 b	39.87 ab
	N240	89.72 b	3.35 b	39.27 b
	N300	103.13 a	3.61 a	40.76 a
2024	N0	65.11 c	3.38 b	42.74 ab
	N60	69.49 bc	3.51 ab	42.56 ab
	N120	82.50 b	3.87 ab	43.33 a
	N180	82.29 b	3.86 ab	42.10 b
	N240	99.21 a	3.83 ab	41.95 b
	N300	105.39 a	4.05 a	42.53 ab

Values followed by different lowercase letters differ significantly at *P <*0.05.

To fit the relationship between seed cotton yield and nitrogen application rate in 2023 and 2024, it was found that the univariate quadratic function provided a better fit to this relationship, and the parameters of the fitting curve were statistically significant. There was a significant positive correlation between seed cotton yield and nitrogen application rate, and the highest yield nitrogen application rate was calculated to be 442.87 kg ha*^−^*^1^. It could be seen that a high nitrogen application rate would improve the yield, but a higher nitrogen application rate did not mean a higher yield. Combined with the price of seed cotton, fertilizer, labor, and other parameters, the quadratic equation of economic benefit with nitrogen application rate was *w* = −0.02656*x*^2^ + 15.22544*x* + 3756.48 (*R*^2^ = 0.581^*^). The optimal economic nitrogen application rate was 286.62 kg ha*^−^*^1^, and the maximum benefit was 5,938.46 yuan ha*^−^*^1^. Although the weather conditions of drought and lack of rain in 2023 had affected the absorption and utilization of nitrogen by cotton, resulting in the incongruity of water and fertilizer in various treatments, and the gap of seed cotton yield between high nitrogen and low nitrogen treatments had further widened, affecting the parameter performance of the fitting curve, the best economic nitrogen application rate fitted was between the nitrogen application levels of the N240 and N300 treatments, and the nitrogen application rate had decreased by 35.28% compared with the highest yield nitrogen application rate. This result showed that the N300 treatment had produced the result of economic benefit decline, and the N240 treatment was the most suitable nitrogen application choice. The maximum nitrogen application rate only pursued yield potential unilaterally, without considering resource input and waste issues. In actual production, the diminishing marginal benefits of nitrogen fertilizer cannot be ignored. When the nitrogen application rate exceeded 240 kg ha*^−^*^1^, the yield increase brought by unit nitrogen fertilizer input significantly decreased, which not only caused waste of nitrogen fertilizer resources but also increased the risk of non-point source pollution in farmlands. According to the calculations in this study, applying 240 kg ha*^−^*^1^ of nitrogen was the economically optimal solution to achieve synergistic optimization of production and ecological benefits while ensuring stable cotton production.

### 3.7 The effect of nitrogen application rate on nitrogen fertilizer use efficiency of cotton

**Figure 7:**
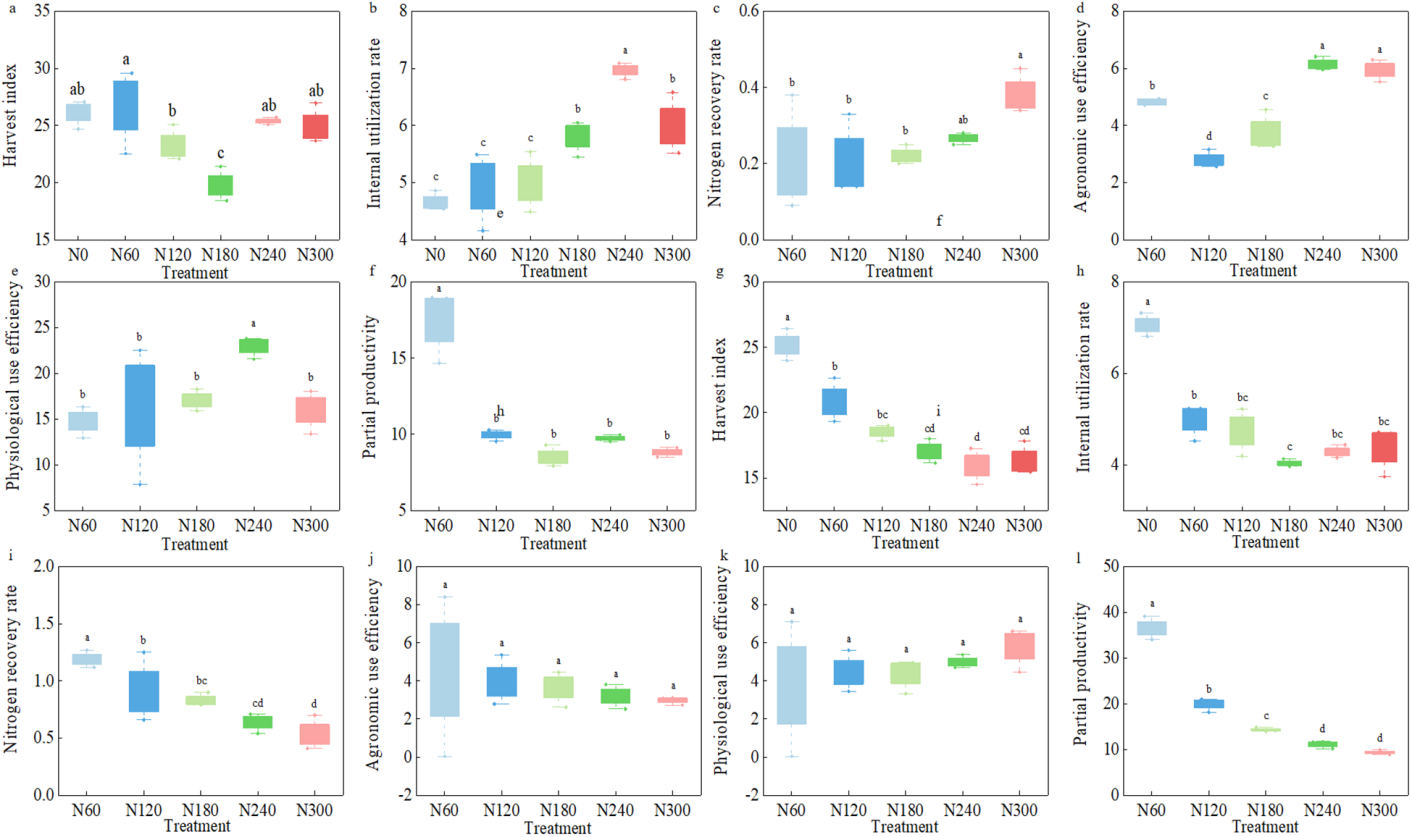
Nitrogen fertilizer use efficiency of cotton under different nitrogen application rates. **(a–f)** The cotton harvest index, nitrogen fertilizer internal use efficiency, nitrogen fertilizer recovery rate, nitrogen fertilizer agronomic use efficiency, nitrogen fertilizer physiological use efficiency, and nitrogen fertilizer partial productivity under different nitrogen application rates in 2023, respectively. **(g–l)** The nitrogen fertilizer use efficiency mentioned above in 2024.

The results in **Figure 7** showed that the partial productivity of nitrogen fertilizer in 2023 and 2024 decreased significantly with the increase of nitrogen application rate. There was no significant difference in the 2-year harvest index, nitrogen recovery rate, nitrogen agronomic use efficiency, and nitrogen partial productivity between the N240 and N300 treatments, and the nitrogen physiological use efficiency and nitrogen internal use efficiency of the N300 treatment in 2023 were significantly lower than those of the N240 treatment by 14.09% and 30.51%, respectively. Compared with the N180 treatment, the N240 treatment significantly increased harvest index, nitrogen internal use efficiency, nitrogen agronomic use efficiency, and nitrogen physiological use efficiency by 28.35%, 19.94%, 64.89%, and 34.71%, respectively. However, there was no significant difference in nitrogen use efficiency between the N240 and N180 treatments in 2024. The results showed that controlling the nitrogen application rate in the range of 180~240 kg ha*^−^*^1^ could effectively play the potential of nitrogen fertilizer application efficiency in cotton fields and improve nitrogen use efficiency.

### 3.8 The effect of nitrogen application rate on cotton fiber quality

The results in **Table 2** indicated that except for the Micronaire value, there were no significant differences in various fiber quality indicators between the N180 and N240 treatments in 2023 and 2024, and there was no significant difference in fiber quality indicators between the N240 and N300 treatments. There was no significant difference in fiber quality indicators among the N0, N60, and N120 treatments. Only in 2023, the average length of the upper half, fracture specific strength, and elongation rate of the N0 treatment were significantly reduced compared to the N240 treatment. The Micronaire values of the N120, N180, and N240 treatments in 2023 and 2024 were all within the range of 3.7~4.2, indicating that the N120, N180, and N240 treatments had stable high-quality Micronaire value cotton fibers. The N300 treatment failed to effectively improve cotton fiber quality, while the N180 and N240 treatments had achieved relatively better fiber quality.

**Table 2:** Cotton fiber quality under different nitrogen application rates.

Year	Treatment	Average length of the upper half (mm)	Fracture specific strength (CN.tex^−1^)	Uniformity index (%)	Macron value	Elongation rate (%)
2023	N0	26.90 b	27.27 c	81.03 abc	3.67 c	4.25 c
	N60	27.08 ab	27.83 bc	80.70 bc	3.67 c	4.32 bc
	N120	26.88 b	28.63 abc	79.85 c	3.75 c	4.40 abc
	N180	27.63 a	29.38 ab	82.28 a	4.07 b	4.35 abc
	N240	27.67 a	29.98 a	81.13 abc	4.13 b	4.53 ab
	N300	27.20 ab	29.35 abc	81.78 ab	4.43 a	4.57 a
2024	N0	28.55 a	28.55 a	85.38 a	4.07 ab	6.30 b
	N60	29.15 a	28.98 a	85.95 a	4.11 ab	6.35 ab
	N120	29.10 a	29.70 a	86.75 a	4.18 a	6.35 ab
	N180	29.30 a	30.70 a	86.72 a	4.18 a	6.45 a
	N240	29.02 a	30.25 a	85.45 a	3.78 b	6.35 ab
	N300	29.45 a	32.08 a	85.88 a	3.83 ab	6.45 a

Values followed by different lowercase letters differ significantly at *P <*0.05.

### 3.9 The relationship between cotton nitrogen nutrition index and relative yield

**Figure 8:**
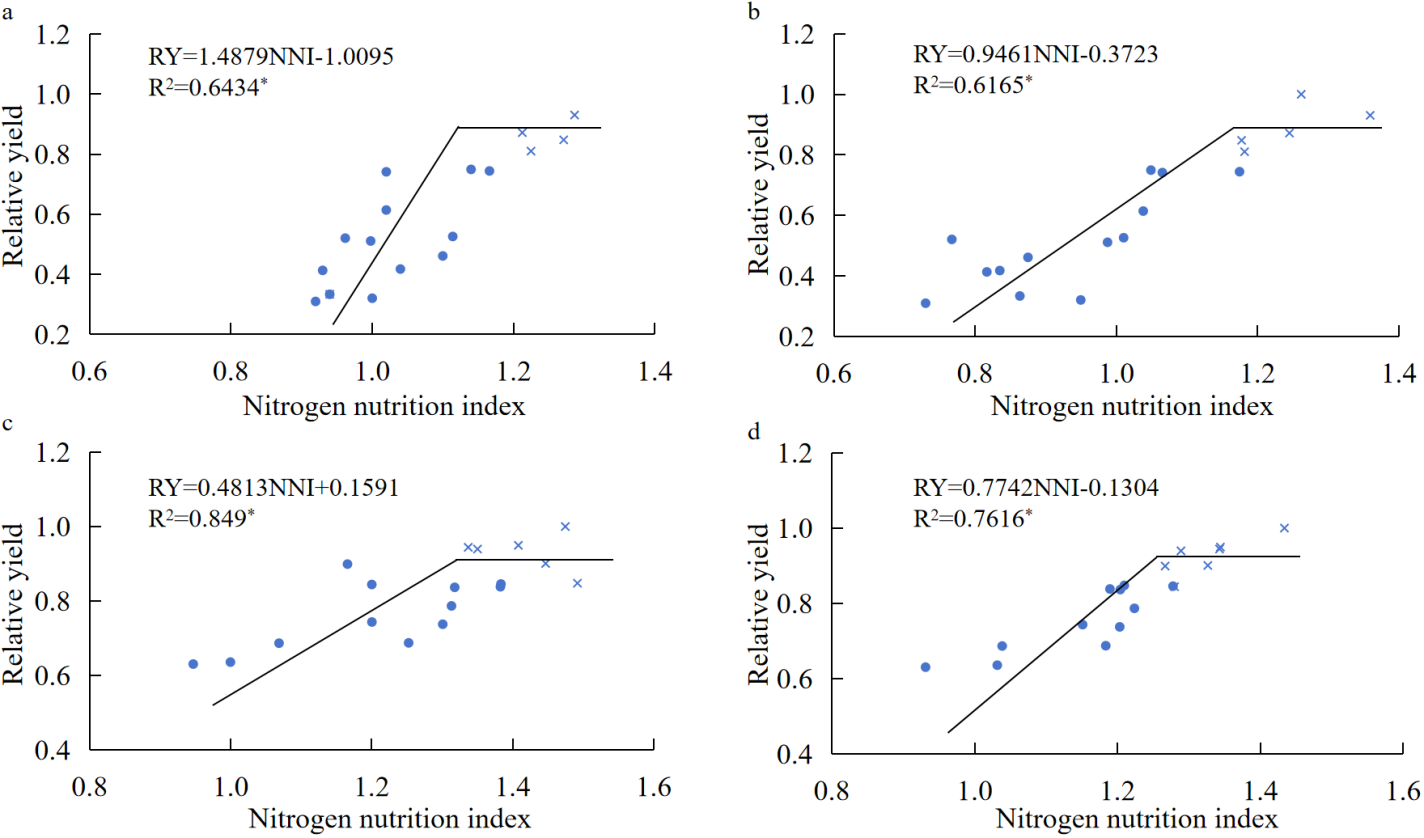
The relationship between the nitrogen nutrition index and relative yield of cotton under different nitrogen application rates. **(a–d)** The fitting relationship between the whole plant NNI in 2023, the reproductive organ NNI in 2023, the whole plant NNI in 2024, and the reproductive organ NNI in 2024 and relative production, respectively.

**Figure 8** shows that the relative yield (RY) of seed cotton was a linear plus platform function relationship with the NNI at the boll opening stage, and the simulation equation based on the whole plant NNI was RY = 1.4879NNI − 1.0095 (*R*^2^ = 0.6434^*^). The simulation equation based on genitalia NNI was RY = 0.9461NNI − 0.3723 (*R*^2^ = 0.6165^*^). In a certain range, RY increased with the increase of NNI; when the NNI of the whole plant and reproductive organs reached 1.22 and 1.17, respectively, RY would reach the peak, which were 0.89 and 0.93, respectively. The fitting equation of the change of RY with the whole plant NNI and genitalia NNI reached a significant level (*P* < 0.05), and the *R*^2^ value of the equation was higher (more than 0.6), indicating that the parameters of the fitting curve of NNI and RY were statistically significant and had a good prediction effect. The results showed that the relative yield performance of cotton could be effectively predicted based on the nitrogen nutrition index of the whole plant and reproductive organs at the boll opening stage. Increasing the nitrogen nutrition index could effectively improve the relative yield, but when the nitrogen nutrition index of the whole plant and reproductive organs was increased to more than 1.22 and 1.17, the seed cotton yield would no longer be effectively increased, which would reduce the utilization efficiency of nutrient resources.

### 3.10 The relationship between biomass, nitrogen nutrition index, nitrogen absorption and utilization, and yield

**Figure 9:**
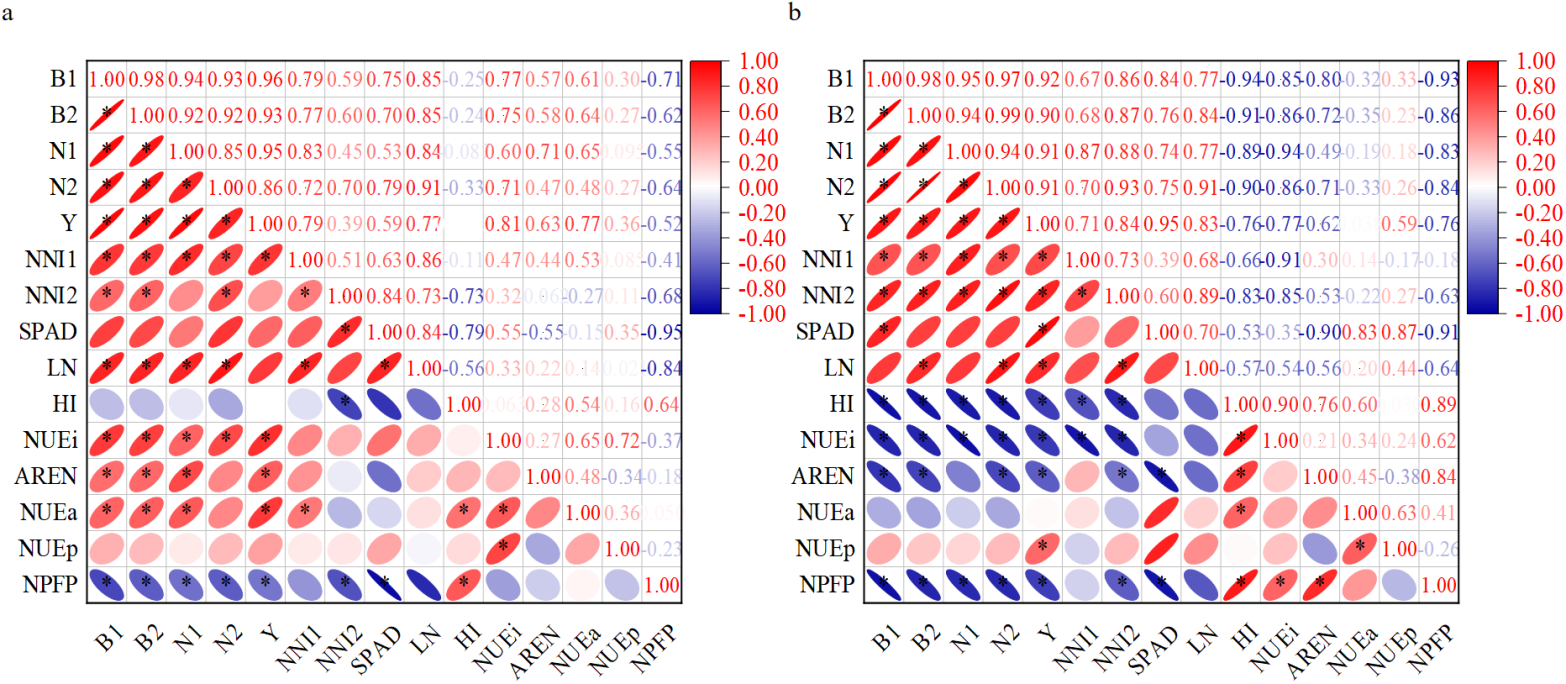
Thermal map of the correlation between NNI, leaf SPAD values, nitrogen concentration, nitrogen uptake, nitrogen fertilizer use efficiency, biomass, and yield. **(a, b)** The correlation between each indicator in 2023 and 2024, respectively. B_1_ and B_2_ respectively represented the biomass of the whole plant population and the biomass of reproductive organs during the boll opening stage; N_1_ and N_2_ respectively represented the nitrogen uptake of the whole plant population and the nitrogen uptake of the reproductive organ population during the boll opening stage; Y represented the seed cotton yield; NNI_1_ and NN_2_ respectively represented the whole plant-based NNI and reproductive organ-based NNI during the boll opening stage; SPAD and LN respectively represented the SPAD value and leaf nitrogen concentration of the inverted four leaves during the boll opening stage; HI, NUE_i_, AREN, NUE_a_, NUE_p_, and NPFP, respectively, represented harvest index, nitrogen internal use efficiency, nitrogen fertilizer recovery rate, nitrogen agronomic efficiency, nitrogen physiological use efficiency, and nitrogen partial factor productivity.

According to the correlation heatmap results in **Figure 9**, there was a significant positive correlation between B_1_, B_2_, N_1_, N_2_, NNI_1_, NNI_2_, and seed cotton yield in 2023 and 2024, with correlation coefficients of 0.96, 0.93, 0.95, and 0.86 in 2023 and 0.92, 0.90, 0.91, and 0.91 in 2024, respectively. There was no significant correlation between NNI_1_ and AREN, NUE_p_, or NPFP in 2023 and 2024. NNI_2_ did not show a significant correlation with NUE_a_ and NUE_p_, but showed a significant negative correlation with HI and NPFP. The correlation coefficients in 2023 were −0.73 and −0.68, respectively, while those in 2024 were −0.83 and −0.63, respectively. There was a significant positive correlation between LN and B_2_, N_2_ in 2023 and 2024, with correlation coefficients of 0.85 and 0.91 and 0.84 and 0.91, respectively. There was a significant positive correlation between nitrogen nutrition index and biomass based on whole plant and reproductive organs over the past 2 years, with a correlation coefficient range of 0.59~0.87. This result indicated that the cotton plant nitrogen nutrition index based on whole plant and reproductive organs during the boll opening stage could be a reliable tool for judging yield performance. Yield increased with the increase of nitrogen nutrition index, but harvest index and nitrogen fertilizer partial productivity gradually decreased with the increase of nitrogen nutrition index. There was a significant correlation between the nitrogen content, dry matter mass, and nitrogen nutrition index of cotton leaves, providing a reliable basis for rapid diagnosis of nitrogen nutrients in the field.

## 4 Discussion

### 4.1 Evaluation of the construction effect of the cotton critical nitrogen concentration dilution curve model in the oil cotton rotation mode

The CNDC is a mathematical model tool for quantifying crop nitrogen demand. In recent years, experimental studies using CNDC as a diagnostic index of nitrogen nutrition status have been widely reported in different crops and regions (Ye et al., 2024; Dongarwar et al., 2023). The NNI derived from the critical nitrogen concentration dilution model could directly and quantitatively judge the nitrogen nutrition of crops (Zhang et al., 2025). In this study, based on the dry matter accumulation, the critical nitrogen concentration dilution curve was constructed to reveal the dynamic coupling relationship between cotton nitrogen absorption and dry matter accumulation. The models based on the whole plant and reproductive organ dry matter were *y* = 26.936*x*^−0.284^ (*R*^2^ = 0.68, *P* < 0.05) and *y* = 23.504*x*^−0.226^ (*R*^2^ = 0.6226, *P* < 0.05), respectively, which reduced the limitations of the previous model only for the cotton monoculture mode and contributed to the improvement of the accuracy of nitrogen nutrition diagnosis of the rapeseed cotton rotation mode in the Yangtze River Basin. The value of a in the model parameters represents the critical nitrogen concentration value when the aboveground dry matter is 1 t·ha*^−^*^1^ (the concentration unit in this study is g kg^−1^), and b is the slope of the critical nitrogen concentration dilution curve. From the perspective of the model construction process, this study obtained the data of dry matter accumulation and nitrogen concentration of cotton at different growth stages under different nitrogen application levels through two consecutive years of field fixed-point experiments and finally determined that the cotton CNDC model under the rapeseed cotton rotation mode was in the form of power function, which was consistent with the previous CNDC model for most conventional crops (corn, rice, wheat, cotton) (Miao et al., 2025; Yu et al., 2023; Yin and Main, 2015), indicating that the power function could reflect the law of crop “growth dilution effect.” However, compared with the parameters of the critical nitrogen concentration dilution curve of monoculture cotton constructed by Chen et al. (2022), the values of parameters a and b of the model were reduced. The reason for this difference might be that, compared with monoculture cotton, the rotation system planted rape in the winter fallow period, and the effect of rape before harvest affected soil nutrients. After harvest, the decomposition of rape plant residues could release nitrogen nutrients to the soil, thus improving the level of soil basic nitrogen at the early stage of cotton growth, resulting in a relatively high nitrogen uptake by cotton under the same dry matter accumulation, resulting in an increase in the slope value of the critical nitrogen concentration model, which was similar to the study of Liu et al. (2023) in the wheat cotton rotation system. The reason why the slope of the dilution curve was different from that of Qin et al. and Zhang et al. might be that straw returning improved the growth environment of cotton roots, promoted nutrient transport to the reproductive organs, and made the slope of the dilution curve relatively slow down (Qin et al., 2025a; Zhang et al., 2023).

The repeatability verification and applicability evaluation of the constructed critical nitrogen concentration dilution curve model are the key to determine whether the model has a stable fitting effect (Clunes and Pinochet, 2020). In this study, complete data (the same test plot, the same field setting, the second consecutive year of test data) were used to verify the CNDC model, and the prediction accuracy of the model was evaluated by goodness of fit (*R*²), RMSE, and standardized nRMSE. In this experiment, the RMSE values of the two models were 2.76 and 3.53, respectively, and the nRMSE values were 15.10% and 14.85%, respectively, which showed that the simulation effect of the CNDC model based on reproductive organs maintained the same good level as that of the CNDC model based on the whole plant, and the nRMSE value was relatively lower, which not only saved time and labor but also improved the stability of the model, and was more suitable for the development of nitrogen nutrition diagnosis tools with the goal of pursuing simplicity and efficiency, which was consistent with the research results of the CNDC model of drip irrigation spring maize (Zhou et al., 2025).

### 4.2 Nitrogen management in cotton field based on nitrogen nutrition diagnosis, yield performance, and nitrogen absorption and utilization

The rapeseed cotton rotation mode is an annual planting system. The nitrogen fertilizer application scheme should take into account yield improvement, nitrogen-efficient utilization, and environmental risk control (Wang et al., 2022). The nitrogen nutrition diagnosis based on the CNDC model quantified the status of nitrogen luxury absorption or nitrogen limitation. The quantitative relationship between NNI diagnosis results and nitrogen absorption and utilization, and yield formation could promote the construction of a precision management system and provide the core quantitative analytical basis for the digital, intelligent, and green development of the cotton industry (Lv et al., 2025; Amrit et al., 2023). In this study, the diagnosis results based on NNI showed that the NNI based on the whole plant and reproductive organs were 1.13~1.39 and 1.12~1.20 in 2023 and 1.05~1.33 and 1.15~1.36 in 2024, respectively, from the beginning of flowering to the boll opening stage. The results showed that the cotton plant with 300 kg ha*^−^*^1^ nitrogen fertilizer was in the state of nitrogen surplus at each growth stage, which was prone to overgrowth, resulting in poor ventilation and light transmission in the field, blocked dry matter transport in the reproductive organs at the late growth stage, and decreased yield. When the nitrogen application rate was 240 kg ha*^−^*^1^, the NNI based on the whole plant and reproductive organs was between 1.02~1.22 and 0.98~1.05 in 2023 and between 1.01~1.33 and 0.99~1.30 in 2024. Compared with the 360 kg ha*^−^*^1^ nitrogen application, the nitrogen supply and demand of cotton was more balanced, the rationality of the population structure was ensured, and the yield also reached the peak (when the nitrogen application rate was 240~300 kg ha*^−^*^1^, the yield and NNI showed a platform relationship), which stimulated the population growth potential, which was consistent with the law of “appropriate nitrogen application promoted yield increase, and excessive nitrogen application led to yield decline” in relevant studies (Wang et al., 2021). Nitrogen absorption and utilization efficiency of plants was the core index to measure the effect of nitrogen application. Research showed that simply pursuing high nitrogen use efficiency might reduce economic benefits. Under the condition that crop yield, quality, and nitrogen input and output were basically balanced, maintaining high nitrogen use efficiency had practical significance (Zurweller et al., 2019). Taking nitrogen nutrition diagnosis as the core index, the accuracy and stability of nitrogen nutrition diagnosis were verified by using yield performance, nitrogen use efficiency, fiber quality performance, and other indicators. By analyzing the differences of boll number, boll weight, and yield stability, the diagnosis results were corrected in reverse, which could achieve a balance between economic benefits and ecological sustainability (Wang et al., 2021; Pendke et al., 2025). This study showed that there was no significant difference in nitrogen use efficiency between the N180 and N240 treatments in 2024, and the NNI index also showed that cotton plants were in a suitable nitrogen state, which was consistent with the nitrogen application range corresponding to the peak yield, and could be used as the optimal nitrogen application range, which was similar to the results of the fertilization test based on the NNI diagnosis in the Xinjiang drip irrigation cotton field (Pei et al., 2023). The internal utilization and physiological utilization of the N300 treatment were significantly lower than those of the N240 treatment. The possible reason was that excessive nitrogen caused high soil nitrogen content, destroyed the rationality of the soil microbial community structure, inhibited cotton root activity and nitrogen absorption capacity at key growth stages, reduced nitrogen conversion efficiency, and failed to significantly improve the nitrogen absorption and yield of cotton plants, which was consistent with the research of Song et al. (2020) in the cotton region of the Yangtze River Basin.

## 5 Conclusions

In this study, the critical nitrogen concentration dilution curve model of cotton in the rapeseed cotton rotation mode was constructed based on the continuous field experiment. The stability and diagnostic effect of the critical nitrogen concentration dilution curve model were verified, and the critical nitrogen concentration dilution curve models based on the biomass of cotton whole plant and reproductive organs were obtained: *y* = 26.936*x*^−0.284^ (*R*^2^ = 0.68, *P* < 0.05) and *y* = 23.504*x*^−0.226^ (*R*^2^ = 0.6226, *P* < 0.05). The model validation parameters RMSE and nRMSE were 2.76 and 3.53 and 15.10% and 14.85%, respectively. The model determination coefficient was statistically significant, and the stability reached a good level. The NNI based on the critical nitrogen concentration dilution curve model could be used to determine the nitrogen nutrition status of cotton plants and could be used as a scientific quantitative tool for diagnosing the nitrogen status of cotton. The nitrogen nutrition index of the cotton plant with nitrogen application of 180~240 kg ha*^−^*^1^ was stable at approximately 1.0, reaching the suitable state of nitrogen. According to the subsection relationship of linear+platform between NNI and RY, when NNI reached 1.22 and 1.17, respectively, RY could obtain the maximum value. The nitrogen nutrition diagnosis model based on the reproductive organs had the advantages of being more labor-saving, efficient, and economical. Nitrogen application of 240 kg ha*^−^*^1^ could be used as the recommended nitrogen application rate for cotton in the rapeseed cotton rotation mode in the Yangtze River Basin.

## Data Availability

The original contributions presented in the study are included in the article/supplementary material. Further inquiries can be directed to the corresponding authors.
